# Improving the Efficiency and Effectiveness of Community Detection via Prior-Induced Equivalent Super-Network

**DOI:** 10.1038/s41598-017-00587-w

**Published:** 2017-03-29

**Authors:** Liang Yang, Di Jin, Dongxiao He, Huazhu Fu, Xiaochun Cao, Francoise Fogelman-Soulie

**Affiliations:** 1 School of Information Engineering, Tianjin University of Commerce, Tianjin, 300134 China; 2 State Key Laboratory of Information Security, Institute of Information Engineering, Chinese Academy of Sciences, Beijing, 100093 China; 3 School of Computer Science and Technology, Tianjin University, Tianjin, 300072 China; 4 School of Computer Software, Tianjin University, Tianjin, 300072 China; 5 Institute for Infocomm Research, Agency for Science, Technology and Research (A*STAR), Singapore, 138632 Singapore

## Abstract

Due to the importance of community structure in understanding network and a surge of interest aroused on community detectability, how to improve the community identification performance with pairwise prior information becomes a hot topic. However, most existing semi-supervised community detection algorithms only focus on improving the accuracy but ignore the impacts of priors on speeding detection. Besides, they always require to tune additional parameters and cannot guarantee pairwise constraints. To address these drawbacks, we propose a general, high-speed, effective and parameter-free semi-supervised community detection framework. By constructing the indivisible super-nodes according to the connected subgraph of the must-link constraints and by forming the weighted super-edge based on network topology and cannot-link constraints, our new framework transforms the original network into an equivalent but much smaller Super-Network. Super-Network perfectly ensures the must-link constraints and effectively encodes cannot-link constraints. Furthermore, the time complexity of super-network construction process is linear in the original network size, which makes it efficient. Meanwhile, since the constructed super-network is much smaller than the original one, any existing community detection algorithm is much faster when using our framework. Besides, the overall process will not introduce any additional parameters, making it more practical.

## Introduction

Community structure is ubiquitous in networks of diverse fields, such as social networks, biological networks and technological networks. It is the foundational component in understanding complex systems. Many downstream tasks, such as link prediction and network embedding, can benefit from the identified community structure. Communities are often considered as subgraphs in which nodes are more tightly connected with each other than with nodes outside the subgraph, albeit the absence of general and widely-accepted definition of community structure across different fields. In the past few decades, many community detection algorithms have been proposed^1–5^. Some of them achieve satisfactory accuracy at the expense of speed, such as nonnegative matrix factorization and modularity maximization based on spectral optimization. However, it has been verified by many recent researches that, when the difference between the number of intra and inter community edges is below a threshold, merely utilizing the network topology is insufficient to correctly identify the communities^6, 7^.

In the past few years, the question of improving community detection performance with additional information besides the network topology has attracted a surge of interest. In real world, additional information, such as node and edge contents, is ubiquitous. Prior information in the form of either node label or pairwise relationship, can be obtained by human labeling depending upon the additional information and domain knowledge. Therefore, many semi-supervised community detection algorithms are designed to combine network topology information and prior information^8–16^. Compared with node labels^8, 9^, pairwise relationships, i.e., must-link and cannot-link constraints, are widely accepted and have the following two advantages. First, they can be obtained more easily. Determining whether two nodes belong to the same community is more readily accessible than identifying which community a node belongs to. Second, pairwise relationships can be used to represent node labels. A pair of nodes with the same node label can be represented through a must-link constraint, while nodes with different labels can be represented through a cannot-link constraint. Therefore, semi-supervised community detection mainly focuses on how to effectively encode pairwise priors so as to produce significant improvement on community detection performance^10–16^.

According to the acquisition strategies for prior information, most semi-supervised community detection algorithms can be divided into two categories, namely passive and active. Given in advance the pairwise prior information, passive semi-supervised community detection designs the algorithm to increase the performance as much as possible^10–14^. For example, Zhang *et al*. modify the network adjacency matrix according to the pairwise prior information and apply existing community detection algorithms to the modified network^11, 12^. Yang *et al*. unify many existing community detection algorithms, including nonnegative matrix factorization and modularity maximization model into a clustering framework in latent space^14^. To force a pair of nodes with must-link to belong to the same community, they encode them to have similar latent space representations by introducing a weighted latent space graph regularization. Different from passive techniques, active semi-supervised community detection techniques, i.e., semi-supervised community detection based on active learning, assume that pairwise prior information is not given in advance and design the algorithm to select pairs of nodes critical for performance improvement, for human labeling^15, 16^. Taking Yang *et al*.’s work as an example, according to the result of nonnegative matrix factorization, they select for human labeling the pair of nodes with largest membership uncertainty, i.e., entropy^15^. Then they modify the adjacency matrix based on the labeled edges. Shi *et al*. jointly consider the maximum uncertainty, maximum impact and minimum redundancy and construct an objective function with submodular and monotonic properties that guarantee the greedy algorithm with a high approximation rate^16^.

Although the above mentioned semi-supervised methods have significantly improved performance, most of them meet common drawbacks that impede the effectiveness and efficiency of community detection. First and foremost, all of them only consider how to improve detection accuracy via prior information, but ignore how to speed up community detection via the priors. Second, most of the algorithms cannot make sure pairwise constraints are met in the detected community structure. For instance, Zhang *et al*. connect the two nodes with must-link and disconnect the nodes with cannot-link^11, 12^. This strategy only increases the probability that nodes with must-link belong to the same community and nodes with cannot-link belong to different communities, but it does not guarantee these constraints are satisfied. Third, most methods require tuning additional parameters to ensure effective encoding of the pairwise prior information. For example, Yang’s unified semi-supervised framework introduces a parameter balancing the impact of the topology information and priors to maximize the performance improvement^14^. All of these drawbacks limit the application of these methods for problems. Besides, most of the semi-supervised algorithms, except Zhang *et al*.^11, 12^, are specific algorithms without generalization to a wide range of unsupervised community detection algorithms. They may only be applied to few unsupervised community detection algorithms and have limitation on benefiting from the development of community detection.

**Figure 1:**
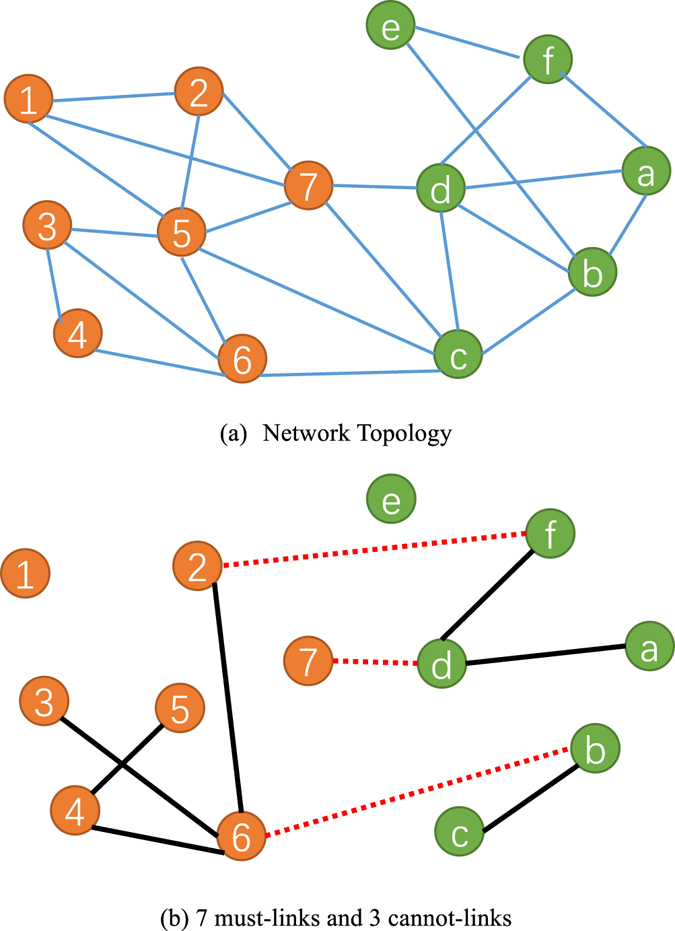
The network topology (**a**) and pairwise (must-link and cannot-link) constraints (**b**). In (**b**) the black solid line and the red dashed line denote the must-link and cannot-link constraints, respectively.

In this paper, to alleviate the afore mentioned issues, especially how to speed up community detection via prior information, we propose a novel semi-supervised community detection framework that can improve both accuracy and speed of existing community detection algorithms through pairwise prior information. The main idea is to construct a super-network based on the network topology (Fig. 1(a)) and pairwise prior information (Fig. 1(b)), which is equivalent to the original network topology with smaller size and tight formulation and preserves the must-link pairwise prior information.

**Figure 2:**
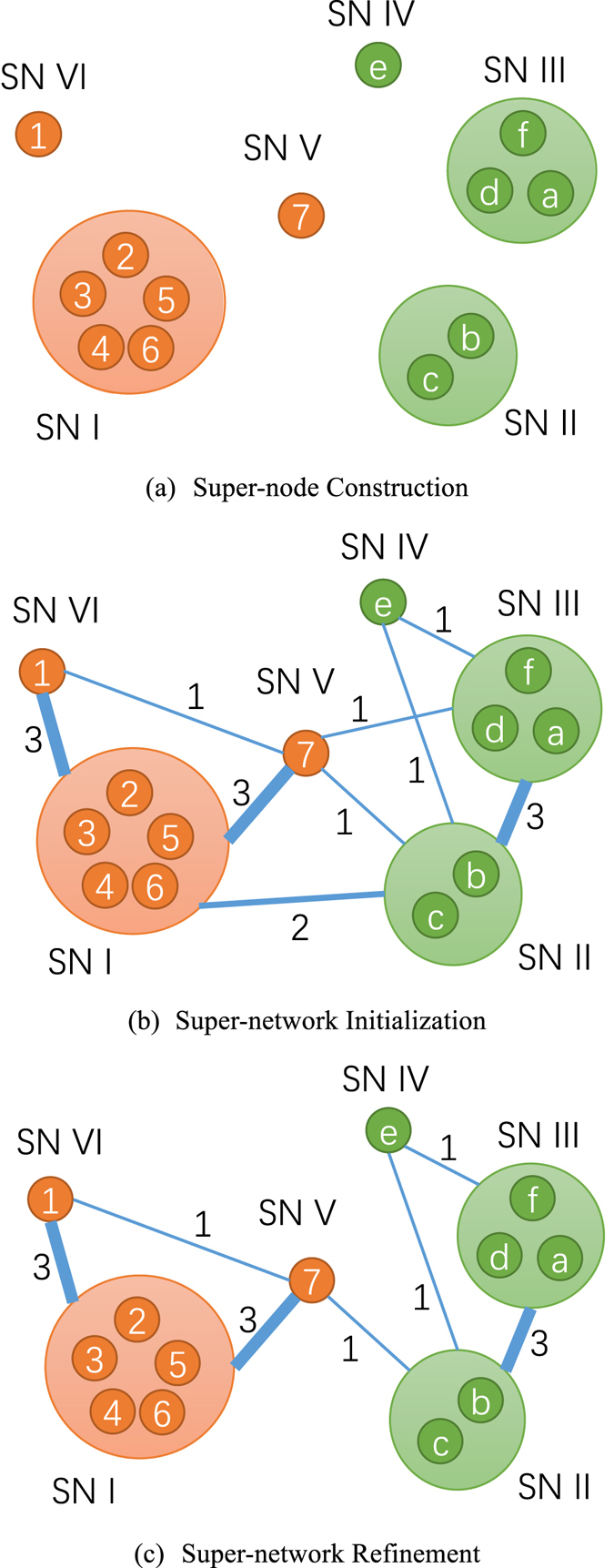
The overall process of the super-network construction.

In the super-network, each super-node consists of a group of nodes in the original network belonging to the same community, and each super-edge between two super-nodes represents the weighted relationship between two super-nodes. The overall process of the proposed framework, which is composed of three steps, is shown in Fig. 2.

In the first step (Fig. 2(a)), the super-nodes are constructed. Specifically, the connected subgraphs, which are the super-nodes in the super-network, are constructed based on the must-link constraints instead of the network topology. This guarantees that nodes in the same connected subgraph (super-node) will belong to the same community. Through this step, the network size, i.e., the number of super-nodes in the super-network, is significantly reduced w.r.t. the original network.

In the second step (Fig. 2(b)), the super-network topology, i.e., super-edges, is initialized. If there is at least one edge between the nodes of two super-nodes, there is a super-edge between the corresponding super-nodes, and the weight of the super-edge should reflect the overall relationship between the two super-nodes. Specifically, the super-edge weight should be in proportion to the total number of edges between the nodes of two super-nodes.

In the third step (Fig. 2(c)), the super-network is refined according to the cannot-link constraints. If there is a cannot-link constraint between the nodes of two super-nodes, the nodes in the two super-nodes must belong to different communities, since the nodes in the same super-node belong to the same community. Therefore, we impose that there is a cannot-link constraint between two super-nodes if there is at least one cannot-link constraint between their nodes. As Zhang *et al*. did, we disconnect the super-edges between super-nodes with cannot-link constraint. Through this step, the number of super-edges in the network can be dramatically reduced.

After the above three steps, the super-network is constructed with *O*(*M* + *N*) time complexity where *N* and *M* are the numbers of nodes and edges, separately. Besides, this process is parameter-free. It means that it does not require to tune any parameter during the process, which makes the process easy to apply in practice.

The constructed super-network effectively integrates the original network topology information and the pairwise prior information. This structure has the following important properties. First, the must-link constraints can be perfectly preserved through the framework, since pairs of nodes with must-link constraints are wrapped within indivisible super-nodes. Second, nodes without any pairwise constraints form super-nodes that only contain themselves, and the relationship between these super-nodes is the same as that between corresponding nodes in the original network. That is, if there is an edge between them in the original network, this edge remains and its weight is 1 in the super-network, while if there is no edge between them in the original network, there does not exist any edge between them in the super-network. Third, the relationship between super-nodes is the combination of topology information and cannot-link constraints. On one hand, since the cannot-link constraint is much stronger than the network topology, if there exists a cannot-link between two nodes in the original network, no super-edge will exist between the corresponding super-nodes. On the other hand, if there does not exit a cannot-link between two nodes, the relationship between the super-nodes containing them is determined by the original network topology and can be seen as the relationship from a more macro perspective. Besides, most of the existing community detection algorithms can be transformed to their semi-supervised versions by applying them to the constructed Super-Network to improve both accuracy and speed. On one hand, since the structure of the super-network is the effective combination of the original network topology information and the pairwise prior information, the performance of community detection should be significantly improved. On the other hand, since the number of super-nodes and super-edges is remarkably reduced, the speed of the community detection should be significantly reduced.

## Results

In this section, we verify the accuracy and the run time of our proposed semi-supervised community detection framework (Super-Network). To this end, we apply two widely-used community detection models, i.e., the nonnegative matrix factorization model with multiplicative updating role^17^ and the modularity maximization model with spectral optimization^18, 19^, to the constructed equivalent super-network. The experiments are conducted on two synthetic network benchmarks and several real world networks. To demonstrate its high accuracy and speed, we take the framework from Zhang *et al*.^12^ as baseline for comparison. Both our approach (Super-Network) and Zhang’s (ModTop) modify the network topology according to the pairwise constraints and can be readily used in many existing community detection methods. ModTop modifies the network topology by adding weighted edges between nodes with the must-link constraint and removing edges with the cannot-link constraint. We set the weight of must-link edge to 1 as Zhang *et al*.^12^. Normalized Mutual Information (NMI)^20^ and run time given in seconds are used to measure the accuracy (the first column in the following result figures) and efficiency (the second column in the following result figures), respectively. Considering that different pairwise constraints with the same amount may cause different performance improvement, we randomly sample 10 groups for each percent and average the resulted accuracy and run time.

### Synthetic Network Benchmarks

We test the proposed Super-Network framework on two synthetic network benchmarks, i.e. Girvan-Newman (GN) benchmark^4^ and Lancichinetti-Fortunato-Radicchi (LFR) benchmark^21^. Each network from GN benchmark is composed of four communities of 32 nodes each. Each node has 16 edges that include Z_*in*_ intra-community edges and Z_*out*_ inter-community edges on average, i.e., Z_*out*_ + Z_*in*_ = 16. Specifically, Z_*out*_ mainly determines the clarity of the community structure, and the task of identifying community structure becomes difficult as Z_*out*_ increases. Compared with GN benchmark, LFR benchmark can generate more flexible networks whose size, distributions of node degree and community sizes and minimum and maximum community size can be specified. Besides, the mixing parameter μ, which is the fraction of inter-community edges and is equivalent to $\frac{{Z}_{out}}{{Z}_{out}+{Z}_{in}}$ in GN, is key to the clarity of community structure and difficulty of identifying communities. In experiments, we set the number of nodes as 1,000, the minimum and maximum community sizes as 10 and 50, the exponent of degree distribution as 2 and that of community size distribution as 1, as did Lancichinetti^21^. To demonstrate the effectiveness and speedup of our framework on the network with diverse community clarities, we vary Z_*out*_ from 7 to 8 for GN networks and μ from 0.7 to 0.75 and 0.8 for LFR networks. On both GN and LFR networks, we apply the nonnegative matrix factorization with multiplicative updating role on the Super-Network and the ModTop networks. We further apply the modularity maximization with spectral optimization on LFR networks, which is much more complicated, to show the generality of our Super-Network framework.

**Figure 3:**
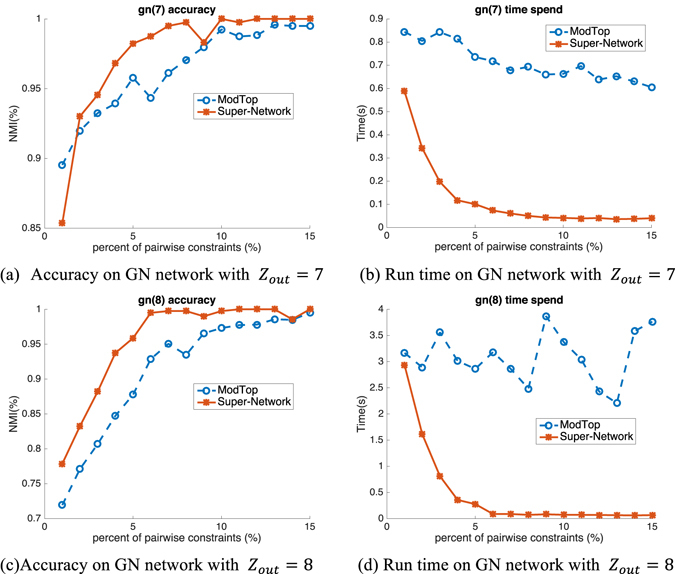
Accuracy (NMI) and run time (spent time in second) on GN benchmark networks based on NMF.

**Figure 4:**
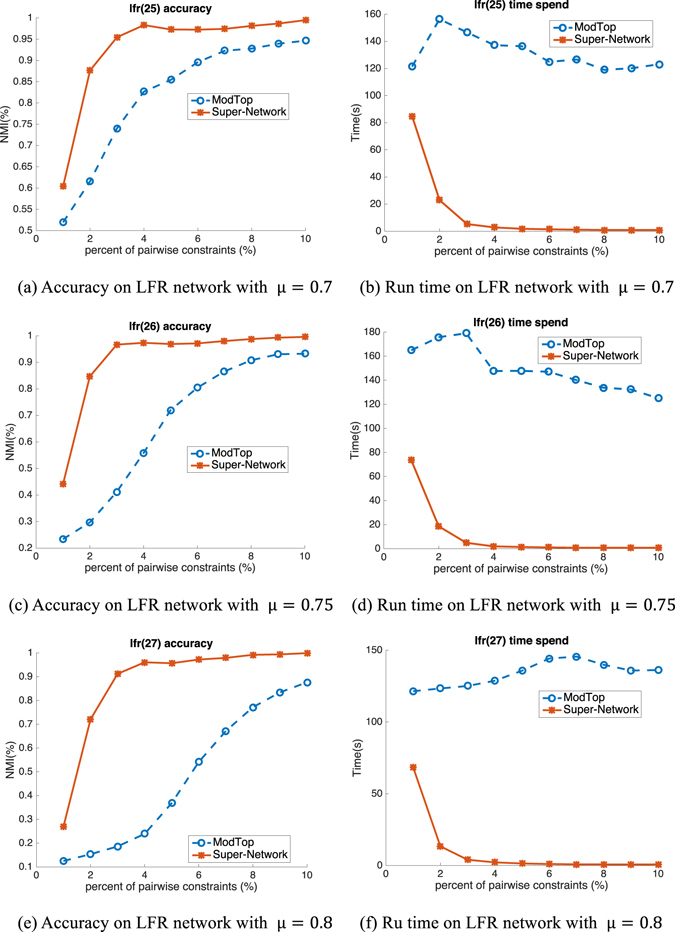
Accuracy (NMI) and run time (spent time in second) on LFR benchmark networks based on NMF.

**Figure 5:**
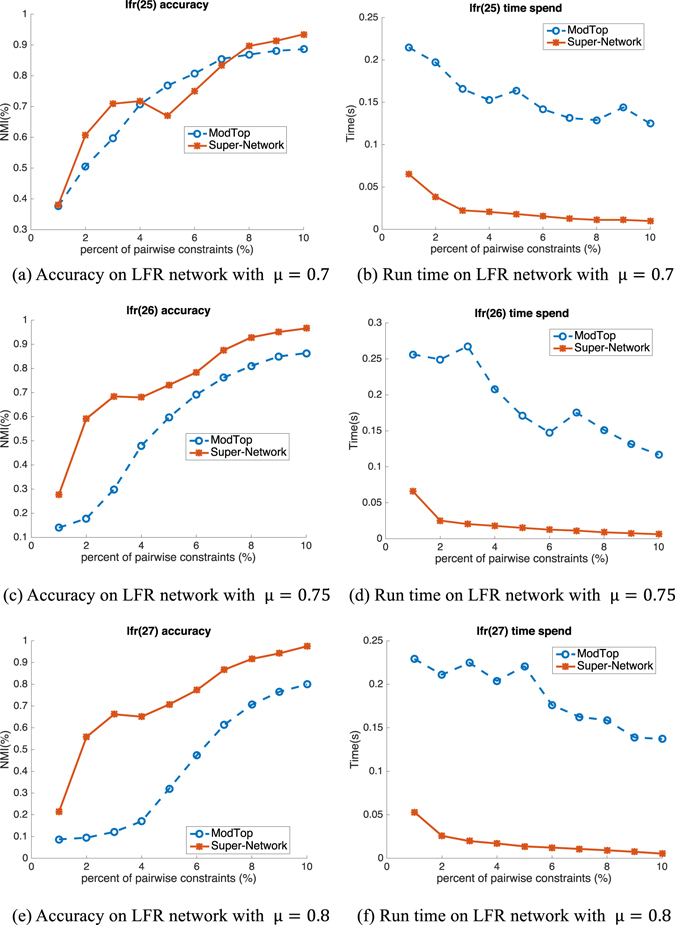
Accuracy (NMI) and run time (spent time in second) on LFR benchmark networks based on modularity maximization with spectral method.

The results are shown in Figs 3, 4 and 5. Figure 3 presents the performance of nonnegative matrix factorization (NMF) on GN networks with Z_*out*_ = 7 (first row) and Z_*out*_ = 8 (second row). From the first column of Fig. 2 which presents the accuracy performance, it can be observed that although both Super-Network and ModTop have improved performance with increasing percent of pairwise constraints, NMF with Super-Network achieves superior NMI accuracy than that with ModTop. Specifically, with 5% pairwise constraints, NMF on Super-Network with Z_*out*_ = 8 achieves 0.96, while that on ModTop only achieves 0.88. This means our Super-Network framework is more effective for pairwise prior information encoding. To evaluate the improvement of speed, we show the run time given in seconds in the second column of Fig. 2. NMF upon Super-Network gets apparent speed advantage compared with NMF on ModTop with increasing percent of pairwise priors. Particularly, taking the original network with Z_*out*_ = 8 and 5% pairwise constraints as an example, time spent on Super-Network (0.28 s) is almost 10 times smaller than ModTop (2.86 s). This illustrates the high efficiency and speed of our proposed Super-Network.

LFR network has a larger structure with more nodes and a topology more complex than GN network, therefore the improvement of accuracy and speed on LFR will be more convincing compared with that on GN. In Figs 4 and 5, we show the performance on LFR network using NMF and modularity maximization with spectral optimization, respectively. In each figure, the three rows are the results on network with μ = 0.7, 0.75 and 0.8, respectively. All of the results illustrate consistent improvement on both accuracy and speed get higher performance upon Super-Network compared to ModTop, especially on speed. For example, on network with μ = 0.75, the time of NMF on Super-Network is about 5 seconds which is 35.8 times faster than ModTop (about 179 seconds). The reason why the time spent on Super-Network extremely decreases is that the size of super-network becomes smaller with decreasing number of nodes and simplified links between nodes. We obtain similar results with higher accuracy and shorter run time as on GN network. In summary, the proposed Super-Network is much more effective and efficient in encoding pairwise constraints on synthetic networks.

### Real-World Large Networks

**Figure 6:**
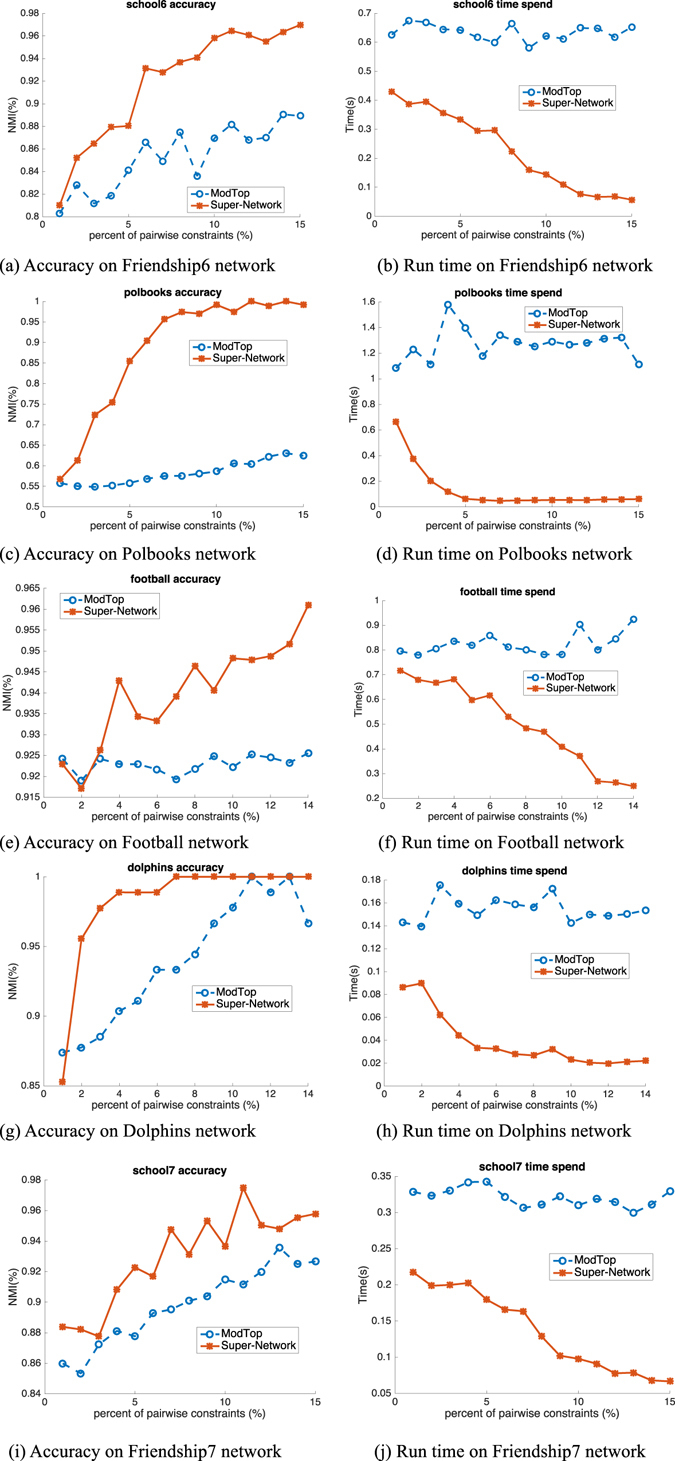
Accuracy (NMI) and run time (spent time in second) on real world networks based on NMF.

**Figure 7:**
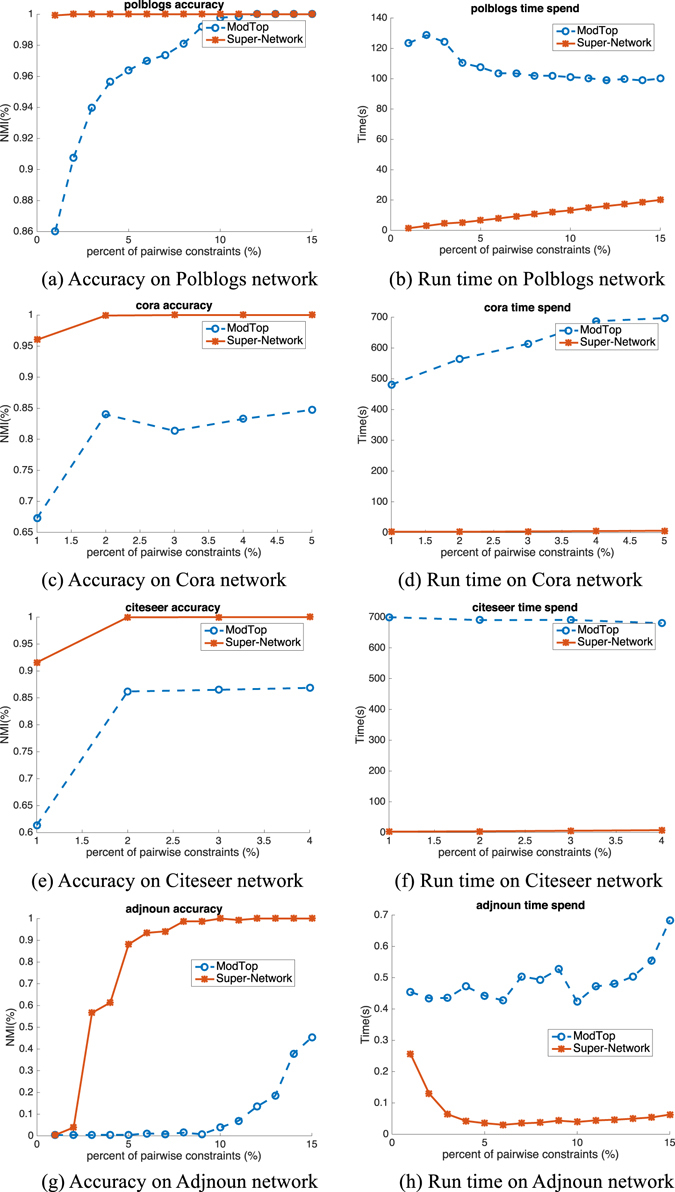
Accuracy (NMI) and run time (spent time in second) on four real world networks based on NMF.

**Figure 8:**
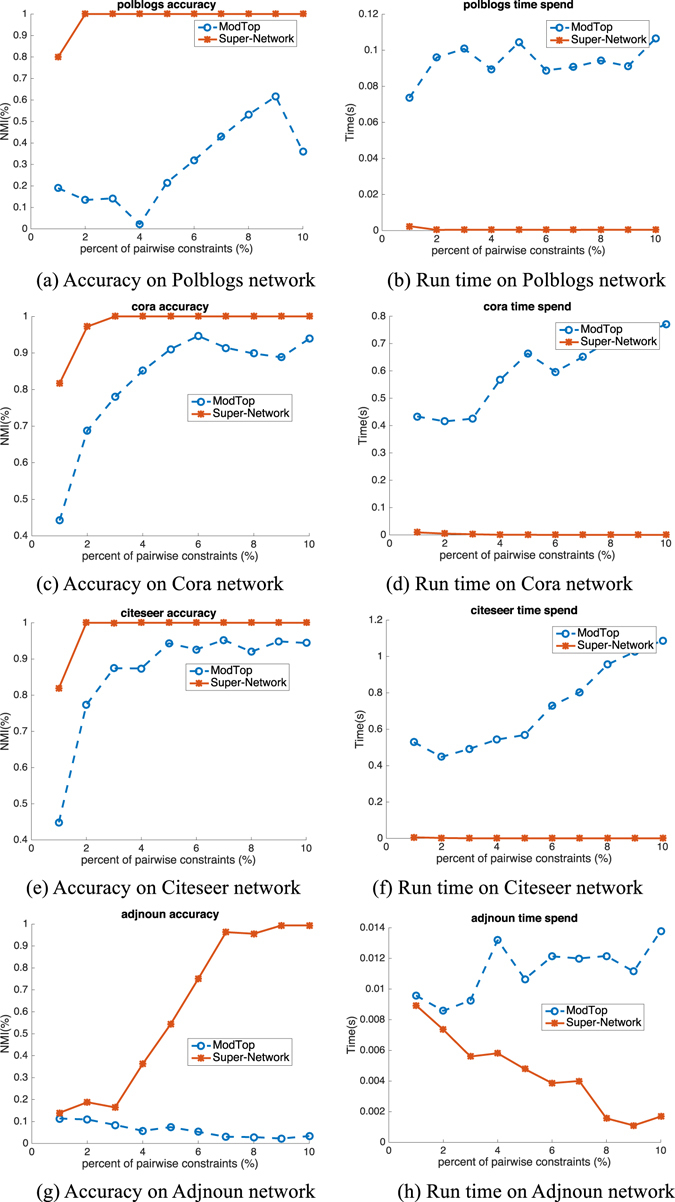
Accuracy (NMI) and run time (spent time in second) on four real world networks based on modularity maximization with spectral method.

We also verify the performance of our proposed Super-Network on 9 real world networks with large variance as shown in Table 1. The number of nodes in networks varies from 62 to 3312. The comparison results are shown in Figs 6, 7 and 8. As done in the synthetic network benchmark, we conduct the NMF on small networks like Dolphins^22^, Football^4^, Friendships^23^ and Polbooks^19^ and both the NMF and modularity maximization with spectral optimization on large networks such as Polblogs^24^, Cora^25^ and Citeseer^25^ etc. The results shown in Fig. 6 illustrate the results on Friendship6 (first row), Polbooks (second row), Football (third row), Dolphins (fourth row) and Friendship7 (fifth row) networks, respectively. The trend of performance improvement and speedup is similar with that found in synthetic networks. Figures 7 and 8 are results of NMF and modularity maximization with spectral optimization, respectively. Both figures show the results on Polblogs (first row), Cora (second row), Citeseer (third row) and Adjnoun^18^ (fourth row) networks. We can find that on Super-Network, both NMF and modularity maximization with spectral optimization are more effective and efficient in encoding the pairwise constraints, and the speedup is more significant on large real world networks than on small ones.

**Table 1:** Real-world networks used here. *N*, *M* and *K* are the numbers of nodes, edges and communities, respectively.

Datasets	*N*	*M*	*K*	Descriptions
Dolphins	62	159	2	Dolphin social network
Football	115	613	12	American College football
Friendship6	69	220	6	High school friendship
Friendship7	69	220	7	High school friendship High school friendship
Polbooks	105	441	3	Books about US politics
Polblogs	1,490	16,718	2	Blogs about US politics
Adjnoum	112	l	2	Word network from novel “David Copperfield”
Cora	2,708	5,429	7	Publication citation dataset from machine learning area
Citeseer	3,312	4,732	6	Publication citation dataset from Citerseer site

### Case Study

**Figure 9:**
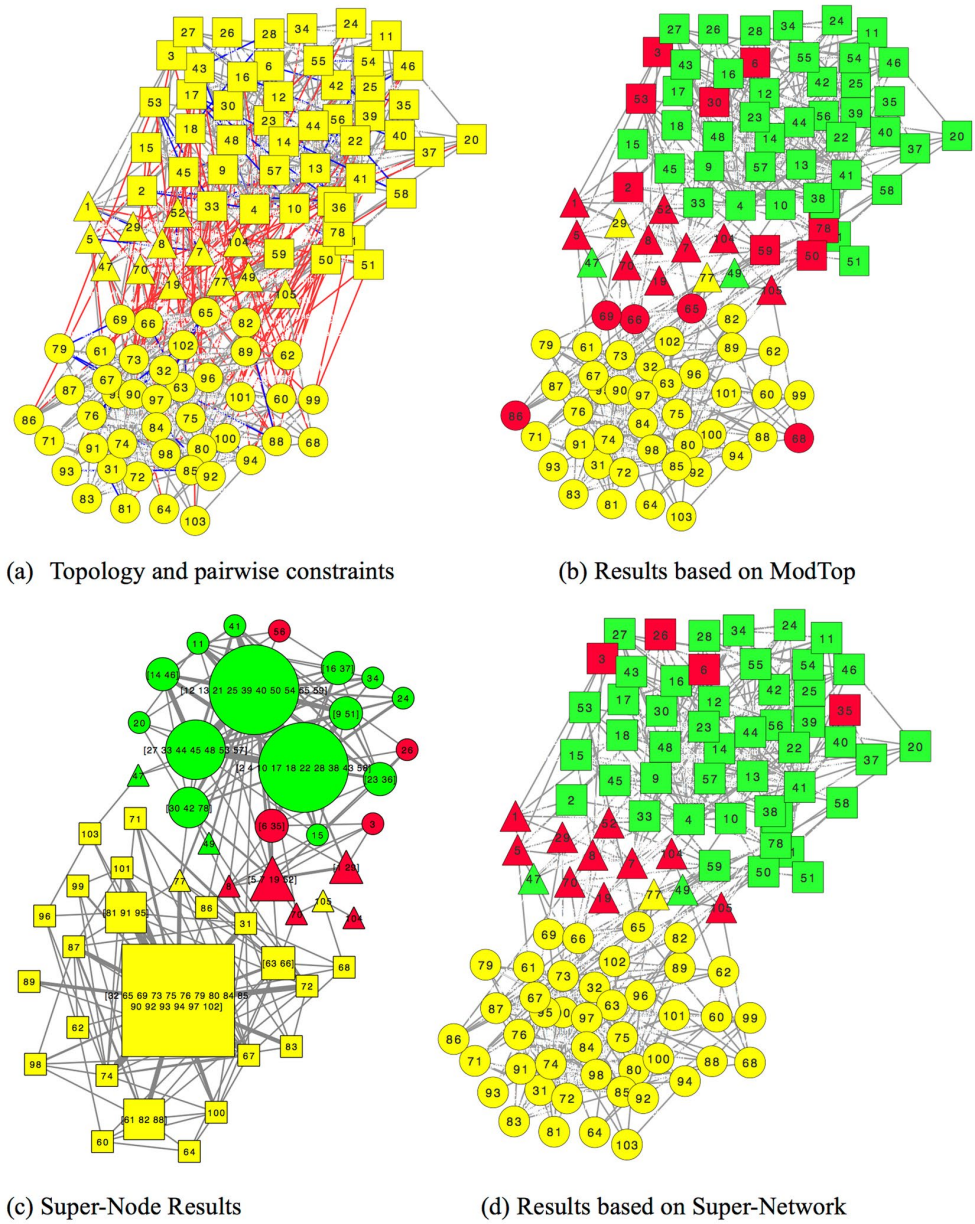
Illustrative example on Polbooks network.

To make the results more intuitive, we carry out a case study on the Polbooks^19^ network. The results are shown in Fig. 9. In Fig. 9(a), we visualize the topology (gray line), 3% must-link constraints (blue line) and 3% cannot-link constraints (red line) in an integrated network. The shape represents ground truth communities while the color represents the detected communities.

NMF on ModTop network is conducted and obtains detection results shown in Fig. 9(b). It is quite obvious that the communities cannot be correctly detected and NMI is 0.55. Figures 9(c) plots the detected results via employing NMF based on super-network. It achieves a higher NMI 0.78 that demonstrates the superiority of detecting communities upon the super-network. The size of a super-node represents the number of nodes contained inside and the width of super-edge indicates its weight. In contrast to the original network in Fig. 9(a), super-network effectively prevents must-link constraints from being broken and greatly decreases the number of nodes and edges which significantly speeds up detection. Taking a super-node containing nodes 30, 42 and 78 for instance, the merged super-node perfectly meets the must-link constraints among these nodes and is indivisible in following steps (nodes 30, 42 and 78 are classified into the same community). Besides, as shown in Fig. 9(c), the size of Super-Network (50) is smaller than that of original network (105), which is the main reason why the algorithms, such as NMF, on Super-Network are faster than that on the original network and ModTop network. In Fig. 9(d) we project the community detection results in Fig. 9(c) upon the original network. The community labels of a super-node and its components are consistent. For example, a super-node consisting of nodes 30, 42 and 78 is classified into “circle” community in the Super-Network, thus nodes 30, 42 and 78 are also classified into “circle” community. Compared with the result in Fig. 9(b), detection result in Fig. 9(d) achieves better performance and effectively corrects the wrongly classified nodes. It strongly demonstrates the effectiveness and efficiency of the proposed super-network framework.

## Discussion

In this paper, we have proposed a novel framework, namely Super-Network, for semi-supervised community detection which can remarkably improve both accuracy and speed of community detection. By constructing the super-nodes as the connected subgraphs determined by the must-link constraints and forming the weighted super-edges based on the original network topology and cannot-link constraints, our framework can effectively and efficiently encode the network topology and pairwise prior information into an equivalent super-network. Since the super-network contains both the topology and pairwise constraint information, many existing unsupervised community detection algorithms can be directly applied to it turning them into semi-supervised algorithms which simultaneously take network topology and pairwise prior information into consideration. From the analysis and experimental results, we find that this semi-supervised super-network framework has the following advantages. First, the effectiveness of encoding pairwise prior is high, since the super-network framework guarantees that the must-link constraints are perfectly preserved. Second, the computing speed and the efficiency are very high, since it significantly reduces the network size only with linear time complexity. Third, the super-network construction process is parameter-free. That is, we do not need to tune any parameter to balance the topology and pairwise priors in practice. In summary, the proposed Super-Network is a general, high speed, effective and parameter-free semi-supervised community detection framework. The proposed Super-Network still has a few weaknesses. First, cannot-link constraints cannot be perfectly guaranteed. That is nodes with cannot-link may be assigned to same community. Second, the inconsistency of the pairwise prior information has not been perfectly solved. We will carry out research on these issues in the future.

## Methods

An undirected network can be represented as a graph *G* = (*V*, *E*) with *N* nodes $V=\{{v}_{1},{v}_{2},\cdots ,{v}_{N}\}$ and *M* edges $E=\{{e}_{ij}\}=\{({v}_{i},{v}_{j})\}$ connecting two nodes *v*_*i*_ and *v*_*j*_, as shown in Fig. 1(a). The network topology can be represented as a binary-valued adjacency matrix $A=\{{a}_{ij}\}\in {\{0,1\}}^{N\times N}$ where *a*_*ij*_ = 1 if there exists an edge between *v*_*i*_ and *v*_*j*_, and *a*_*ij*_ = 0 otherwise. The must-link and cannot-link prior information can be modeled as sets of pairs $ML=\{({v}_{i},{v}_{j})\}$ and $CL=\{({v}_{i},{v}_{j})\}$, respectively, as shown in Fig. 1(b). For convenience, we also represent cannot-link constraints by a binary-valued matrix $C=\{{c}_{ij}\}\in {\{0,1\}}^{N\times N}$ where *c*_*ij*_ = 0 if there is a cannot-link constraint between *v*_*i*_ and *v*_*j*_, and *c*_*ij*_ = 0 otherwise. Besides, we assume that the number of communities *K* is known as a prior.

### Super-Network Construction

To simultaneously improve the accuracy and speed of community detection, we construct an equivalent super-network ${G}^{s}=({V}^{s},{E}^{s})$.${V}^{s}=\{{v}_{1}^{s},{v}_{2}^{s},\cdots ,{v}_{{N}^{s}}^{s}\}$ is the set of *N* ^*s*^ super-nodes (SNs) each of which contains at least one node in the network *G*. ${E}^{s}=\{{e}_{ij}^{s}\}=\{({v}_{i}^{s},{v}_{j}^{s})\}$ is the set of *M* ^s^ super-edges (SEs) each of which connects two super-nodes ${v}_{i}^{s}$ and ${v}_{j}^{s}$ in *V* ^*s*^. The super-network requires to be effective and efficient in integrating the network topology information and the pairwise prior information. The overall process consists of three steps, namely super-node construction, super-network initialization and refinement, as shown in Fig. 2.

### Super-node Construction

To guarantee that the must-link constraints are perfectly met, a straightforward idea is to make the nodes with must-link constraints indivisible. Consequently, we merge the nodes belonging to the same community into a super-node as shown in Fig. 2(a). For example, if we know nodes f and d belong to the same community and nodes d and a belong to the same community, nodes f, d and a must belong to the same community. Thus, we merge them into a super-node SN III. If a node is not included in any must-link constraints, it forms itself as a super-node, such as node 1, 7 and e. This process is equivalent to constructing a connected subgraph according to the must-link constraints. In the following steps, we treat each super-node as an indivisible unit for community detection. The advantages of super-node construction are twofold. On the one hand, the must-link constraints are perfectly met since the super-nodes are indivisible. On the other hand, the number of super-nodes *N* ^*s*^ in the super-network *G* ^*s*^ is much less than that of nodes *N* in the original network *G*. Thus, the community detection algorithms on *G* ^*s*^ are much faster than on *G*. These two advantages make our framework effective and efficient on must-link encoding.

### Super-network Initialization

Given the constructed super-nodes, we initialize the super-network topology, i.e. super-edges, which represents the relationship between super-nodes. Since each super-node consists of multiple nodes from the original network, the relationship between two super-nodes should reflect the summarization of relationships between the nodes of the two super-nodes. Intuitively, if there exists no connection between the nodes of the two super-nodes, we add no super-edge between these two super-nodes. Otherwise, we add a super-edge between these two super-nodes, and set the weight of the super-edge as the total number of connections between the nodes of the two super-nodes. For example, as shown in Fig. 2(b), there are 3 connections between the nodes of SN II and SN III, i.e., the connection between nodes a and b, between nodes b and d and that between nodes c and d. Therefore, the weight of the super-edge between SN II and SN III is equal to 3.

Formally, we represent the assignment of nodes to super-nodes by a binary matrix ${\bf{B}}=\{{b}_{pj}\}\in {\{0,1\}}^{N\times {N}^{s}}$, where *b*_*pj*_ = 1 if node *v*_*p*_ belongs to super-node ${v}_{j}^{s}$ and *b*_*pj*_ = 0 otheriwse. Then ${b}_{pi}{a}_{pq}{b}_{qj}=1$ if and only if *b*_*pi*_ = 1, *b*_*qj*_ = 1 and *a*_*pq*_ = 1, which means node *v*_*p*_ belongs to super-node ${v}_{i}^{s}$, node *v*_*q*_ belongs to super-node ${v}_{j}^{s}$ and there is a link between nodes *v*_*p*_ and *v*_*q*_, respectively. It indicates that if there is a link between nodes *v*_*p*_ (from super-node ${v}_{i}^{s}$) and *v*_*q*_ (from super-node ${v}_{j}^{s}$), ${b}_{pi}{a}_{pq}{b}_{qj}=1$, and $\sum _{p=1}^{N}\sum _{q=1}^{N}{b}_{pi}{a}_{pq}{b}_{qj}$ is the total number of links between super-nodes ${v}_{i}^{s}$ and ${v}_{j}^{s}$. Therefore, we specify the adjacency matrix of the super-network as ${{\bf{A}}}^{{\boldsymbol{s}}}=\{{a}_{ij}^{s}\}\in {\{0,1\}}^{{N}^{s}\times {N}^{s}}$ where ${a}_{ij}^{s}=\sum _{p=1}^{N}\sum _{q=1}^{N}{b}_{pi}{a}_{pq}{b}_{qj}$, i.e.,

$${{\bf{A}}}^{{\boldsymbol{s}}}={{\bf{B}}}^{{\boldsymbol{T}}}{\bf{AB}}.$$

Although the elements on the diagonal of **A** ^***s***^ denote the number of links in each super-node, to make the structure of **A** ^***s***^ to be similar to **A**, they are set to 0.

By doing so, the topology of the super-network is initialized and can be regarded as a view of the original network topology from the macro viewpoint of super-nodes. The relationship between two super-nodes, each of which only consists of one node from the original network, is defined as the same as that between these two nodes in the original network. For example, if there exists an edge between nodes 1 and 7 in the original network, a super-edge with weight 1 is settled between SN V (only consists node 1) and SN VI (only consists node 7).

### Super-network Refinement

After the above two steps, the super-network is constructed by considering the network topology and must-link constraints. In this step, we refine the super-network topology via cannot-link constraints. This step is composed of two sub-steps, i.e., constructing cannot-link constraints between super-nodes and applying super-node cannot-link to super-network topology.

First, since nodes in the same super-node must belong to the same community, if there is a cannot-link constraint between two nodes from different super-nodes, all the nodes in these two super-nodes must belong to different communities. Therefore, we add a cannot-link constraint between these super-nodes in the super-network, if there exists at least one cannot-link constraint between the nodes from these two super-nodes. Formally, as in the super-network initialization step, we obtain the cannot-link constraints for super-nodes as

$${{\bf{C}}}^{{\boldsymbol{s}}}=\{{c}_{ij}^{s}\}={{\bf{B}}}^{{\boldsymbol{T}}}{\bf{CB}},$$

where **C** is the original cannot-link constraint matrix and **B** is the relationship matrix considering nodes and super-nodes. The element ${c}_{ij}^{s}$ is the total number of cannot-link constraints between the nodes in super-nodes ${v}_{i}^{s}$ and ${v}_{j}^{s}$. For example, since there is a cannot-link between nodes 6 and b as shown in Fig. 1(b), we add a cannot-link constraint between SN I (contains node 1) and SN II (contains node b).

Second, given the cannot link constraints for super-nodes, motivated by Zhang *et al*., we modify the super-network topology by removing super-edges between the super-nodes with cannot-link constraints. Since the cannot-link constraint is much stronger than the network topology, it will be implemented no matter how large the weight of the super-edge is. Formally, we refine the initialized super-network topology **A** ^***s***^ with super-node cannot-link constraints **C** ^***s***^ as

$${{\bf{A}}}^{{\boldsymbol{s}}}=\,{\rm{\max }}({{\bf{A}}}^{{\boldsymbol{s}}}-{\rm{\alpha }}{{\bf{C}}}^{{\boldsymbol{s}}},\,{\bf{0}}),$$

where α is a large parameter that makes the cannot-link constraint suppress the adjacency matrix **A** ^***s***^ and set to be equal to the largest element in **A** ^***s***^ in general. For example, though the super-edge weight between SN I and SN II is 2, we remove this super-edge since there exists a cannot-link between them as shown Fig. 2(c).

After the above three steps, i.e., super-node construction, super-network initialization and refinement, our super-network is effectively constructed by integrating the network topology information with pairwise constraints. Subsequently, we can apply many widely-used community detection algorithms, including symmetric nonnegative matrix factorization method or modularity maximization model, on the super-network to detect the communities. Due to the smaller numbers of super-nodes and super-edges, the algorithms on the super-network will be much faster than on the original network.

### Complexity Analysis

Since the equivalent super-network construction consists of three components, we analyze their complexities one by one. First, the complexity of super-node construction requires *O*(*ML* + *N*), where *N* and *ML* are the numbers of nodes and must-link constraints, respectively, according to the complexity of connected subgraphs construction. Second, in super-network initialization, we need to obtain the corresponding super-nodes of each node and accumulate the total number of edges between two super-nodes. These two sub-steps respectively require O(*N*) and O(*M*) operations where *M* is the number of edges. Third, in the super-network refinement, disconnecting super-nodes with cannot-link constraint consumes O(*CL*) operations where *CL* is the number of cannot-link constraints. Therefore, the overall complexity is *O*(*M* + *N* + *ML* + *CL*). Since the number of pairwise constraints is less than that of edges, the overall complexity is reduced to *O*(*M* + *N*). As a result, the process of super-network construction will not increase the complexities of most existing community detection methods whose complexities are higher than or equivalent to *O*(*M* + *N*).

### Inconsistency of Pairwise Prior Information

In the previous discussion, we have obtained the pairwise prior information from ground truth and is consistent. However, in practice it may be inconsistent. For example, if the prior information indicates there are a must-link between nodes f and d, a must-link between nodes f and 7 and a cannot-link between nodes 7 and d, we may find there is inconsistency between these constraints. Here, we consider how to extend the Super-Network framework to incorporate the inconsistent pairwise prior information. The solution is to consider cannot-link with higher priority than must-link. That is to retain the cannot-link constraint and treat the corresponding must-link as normal link. Therefore, in the above inconsistency example, we take the must-link between nodes f and d and the must-link between nodes f and 7 as normal links, and remain the cannot-link between nodes 7 and d. This treatment is based on the following observations. First, there must be at least one wrong constraint between these nodes, thus all the corresponding constraints are not credible. Second, must-link can be perfectly guaranteed by our framework, thus if some of them are not credible, we should weaken the strength of the must-link constraints. Third, by treating the must-link as normal link, the probability of assigning corresponding nodes to the same community will increase. However, it is also possible that they be assigned to different communities according to the network topology. Fourth, although the probability of assigning corresponding nodes with cannot-link to different communities will increase, it is also possible that they be assigned to the same community according to the network topology. Therefore, we keep the cannot-link constraint. We can find that this treatment is a compromise approach to balance the positive and negative impacts of untrusted pairwise prior information.
